# The extent of Skeletal muscle wasting in prolonged critical illness and its association with survival: insights from a retrospective single-center study

**DOI:** 10.1186/s12871-025-03142-7

**Published:** 2025-05-26

**Authors:** Johannes Kolck, Clarissa Hosse, Uli Fehrenbach, Nick-Lasse Beetz, Timo Alexander Auer, Christian Pille, Dominik Geisel

**Affiliations:** 1 Department of Radiology, Charité - Universitätsmedizin Berlin, Berlin, Germany; 2 Berlin Institute of Health at Charité - Universitätsmedizin Berlin, Berlin, Germany; 3 Department of Anesthesiology and Intensive Care Medicine| CCM| CVK, Charité– Universitätsmedizin Berlin, Berlin, Germany; 4 Department of Radiology, Charité– Universitätsmedizin Berlin, Corporate Member of Freie Universität Berlin and Humboldt-Universität zu Berlin, Augustenburger Platz 1, 13353 Berlin, Germany

**Keywords:** Critical care, Acute pancreatitis, COVID-19, Muscle wasting, Artificial intelligence, Computed tomography

## Abstract

**Objective:**

Muscle wasting in critically ill patients, particularly those with prolonged hospitalization, poses a significant challenge to recovery and long-term outcomes. The aim of this study was to characterize long-term muscle wasting trajectories in ICU patients with acute respiratory distress syndrome (ARDS) due to COVID-19 and acute pancreatitis (AP), to evaluate correlations between muscle wasting and patient outcomes, and to identify clinically feasible thresholds that have the potential to enhance patient care strategies.

**Materials and methods:**

A collective of 154 ICU patients (100 AP and 54 COVID-19 ARDS) with a minimum ICU stay of 10 days and at least three abdominal CT scans were retrospectively analyzed. AI-driven segmentation of CT scans quantified changes in psoas muscle area (PMA). A mixed model analysis was used to assess the correlation between mortality and muscle wasting, Cox regression was applied to identify potential predictors of survival. Muscle loss rates, survival thresholds and outcome correlations were assessed using Kaplan-Meier and receiver operating characteristic (ROC) analyses.

**Results:**

Muscle loss in ICU patients was most pronounced in the first two weeks, peaking at -2.42% and − 2.39% psoas muscle area (PMA) loss per day in weeks 1 and 2, respectively, followed by a progressive decline. The median total PMA loss was 48.3%, with significantly greater losses in non-survivors. Mixed model analysis confirmed correlation of muscle wasting with mortality. Cox regression identified visceral adipose tissue (VAT), sequential organ failure assessment (SOFA) score and muscle wasting as significant risk factors, while increased skeletal muscle area (SMA) was protective. ROC and Kaplan-Meier analyses showed strong correlations between PMA loss thresholds and survival, with daily loss > 4% predicting the worst survival (39.7%).

**Conclusions:**

To our knowledge, This is the first study to highlight the substantial progression of muscle wasting in prolonged hospitalized ICU patients. The mortality-related thresholds for muscle wasting rates identified in this study may provide a basis for clinical risk stratification. Future research should validate these findings in larger cohorts and explore strategies to mitigate muscle loss.

**Clinical trial number:**

Not applicable.

**Supplementary Information:**

The online version contains supplementary material available at 10.1186/s12871-025-03142-7.

## Introduction

Muscle wasting in critically ill patients, particularly during prolonged hospitalization, is a significant barrier to recovery and long-term outcomes. Intensive Care Unit-Acquired Weakness (ICUAW), a common complication in these patients, is characterized by profound loss of muscle mass and function [1–3]. Muscle wasting often begins rapidly after ICU admission and progressively worsens over time [4, 5]. The severity of muscle wasting is closely related to the length of hospital stay and is particularly severe in patients with sepsis [6, 7]. Although early muscle loss in critically ill patients is well documented, the understanding of long-term muscle loss remains incomplete.

In this study, we used AI-driven segmentation of clinically indicated computed tomography (CT) scans to perform long-term monitoring of muscle changes in two homogeneous cohorts of critically ill patients. The first cohort included individuals with acute respiratory distress syndrome (ARDS) due to COVID-19 pneumonia, while the second cohort included patients with severe acute pancreatitis (AP) [8, 9]. Our analysis aimed to characterize long-term muscle loss, evaluate its relationship with patient outcomes, and identify clinically applicable thresholds that have the potential to enhance patient care and management strategies in the intensive care unit.

## Materials and methods

### Study design and cohort

This retrospective study analyzed changes in psoas muscle area (PMA) in patients admitted to the ICU for SARS-CoV-2 infection or acute pancreatitis (AP). The study was approved by the Institutional Review Board (internal registration number: EA4/152/20) and adhered to the tenets of the Declaration of Helsinki. Informed consent was waived by the IRB. All of the here included patients were part of two previous assessments [8, 9] and were enrolled between 2012 and 2022. While the cohorts represent two different entities, both demonstrated similar severity, characterized by prolonged ICU stays and frequent imaging, which made them comparable in terms of the key study parameters. By pooling the previously published ICU cohorts, we aimed to enhance statistical power, improve generalizability, and ensure more precise estimates. In contrast to previous studies, this investigation focuses on generating clinically applicable cut-offs for muscle wasting and on presenting the timing of muscle wasting in a new manner. Inclusion criteria required patients to be adults with a minimum ICU stay of 10 days and at least three abdominal CT scans obtained during hospitalization. The cohort included 154 patients with a total of 988 CT scans. Patient consent was waived by the ethics committee.

### Body composition analysis and assessment of muscle decay rates

Tissue compartment quantification was performed using an AI-based automated image segmentation tool integrated into the hospital’s Picture Archiving and Communication System (PACS) software (Visage version 7.1, Visage Imaging GmbH, Berlin, Germany). This tool, previously validated in other studies [10], identified the third lumbar vertebra (L3) level and segmented tissues into subcutaneous fat (SAT), skeletal muscle area (SMA), visceral fat (VAT) and psoas muscle area (PMA). The calculated areas were expressed in square centimeters (cm²). Manual corrections were made by an experienced radiologist when necessary.

To assess muscle loss rates, relative muscle loss per day was calculated for the entire hospital stay by determining the difference in psoas muscle area (PMA, cm²) between the first and last CT scan, divided by the number of intervening days. Relative muscle loss per day was derived by normalizing the absolute daily PMA loss to the baseline PMA of the first scan. In addition, the maximum muscle loss between two consecutive scans and the loss between the first two scans were calculated using the same approach. As a complementary metric, the percentage of total relative muscle loss from the first to the last scan was reported, regardless of the time interval.

### Statistics

Descriptive statistics were presented as medians with interquartile range (IQR). Muscle loss rates and clinical variables were compared between survivors and non-survivors using the Mann-Whitney U test, due to the non-normal distribution of the data. Homogeneity analysis was conducted to ensure pooling of data was adequate. A linear mixed model analysis was used to assess the correlation of mortality and repeated PMA measurements, allowing for the inclusion of unevenly spaced time points. Backward stepwise elimination was used to select the relevant variables for Cox regression from an initial set that included SMA, VAT, SAT, PMA, age, BMI, gender, comorbidities, days of mechanical ventilation, days of ICU stay, first and highest SOFA score, Charlson comorbidity index, presence of overweight and sarcopenia, as well as muscle loss parameters. Cox regression analysis was applied to assess the independent relationships between the selected variables and mortality. Loss rates were analyzed using receiver operating characteristic (ROC) analysis and Youden index to define a threshold for survival prediction. Kaplan-Meier curves were generated to assess survival probabilities for each threshold. Maximum loss was categorized into increments of 0–2%, 2–4%, > 4% PMA loss per day, Kaplan-Meier were equally calculated for these three categories.

Overweight and obesity were defined using internationally recognized BMI thresholds (BMI > 25 kg/m² for overweight, BMI > 30 kg/m² for obesity). Sarcopenia was defined using gender-specific cut-offs: Skeletal muscle index (SMI) < 34.3 cm² for women and < 45.4 cm² for men, according to the established literature [11]. A p-value of less than 0.05 was considered statistically significant. Statistical analyses were performed using SPSS software version 29.0 (IBM, New York, USA) and Jamovi Version 2.3 (The jamovi project, Sydney, Australia).

## Results

### Population characteristics and hospitalization

The study included 154 patients (112 men and 42 women) admitted to the ICU, 100 of them due to acute pancreatitis (AP) and 54 with severe ARDS due to SARS-CoV-2 infection. Median age was 59 (50–68) years, median BMI was 27.5 (23.77–30.04) kg/m². 96 patients (62.3%) were rated as overweight, 42 (27.3%) were categorized as obese. Chronic preconditions were present in 73.4% of all patients, with arterial hypertension being the most common (74/154 patients), followed by cardiovascular diseases (44/154), diabetes (28/154), and pulmonary conditions (22/154). Mean Charlson Comorbidity Index was 1 (0–2). The median hospital length of stay across all patients was 90.5 (45.75–135.25) days, including 61.05 (26.35–95.65) days in the ICU and a median of 44.5 (14.6–74.4) days of invasive mechanical ventilation (IMV). The median Sequential Organ Failure Assessment (SOFA) score at ICU admission was 10 (7–13), the highest SOFA score during hospitalization was 14 (12–16). Overall survival was 57.80%. Compared with survivors, non-survivors were significantly older, had shorter hospital and ICU stays, significantly higher peak SOFA scores and higher rates of muscle wasting. These results are summarized in Table 1.

**Table 1:**
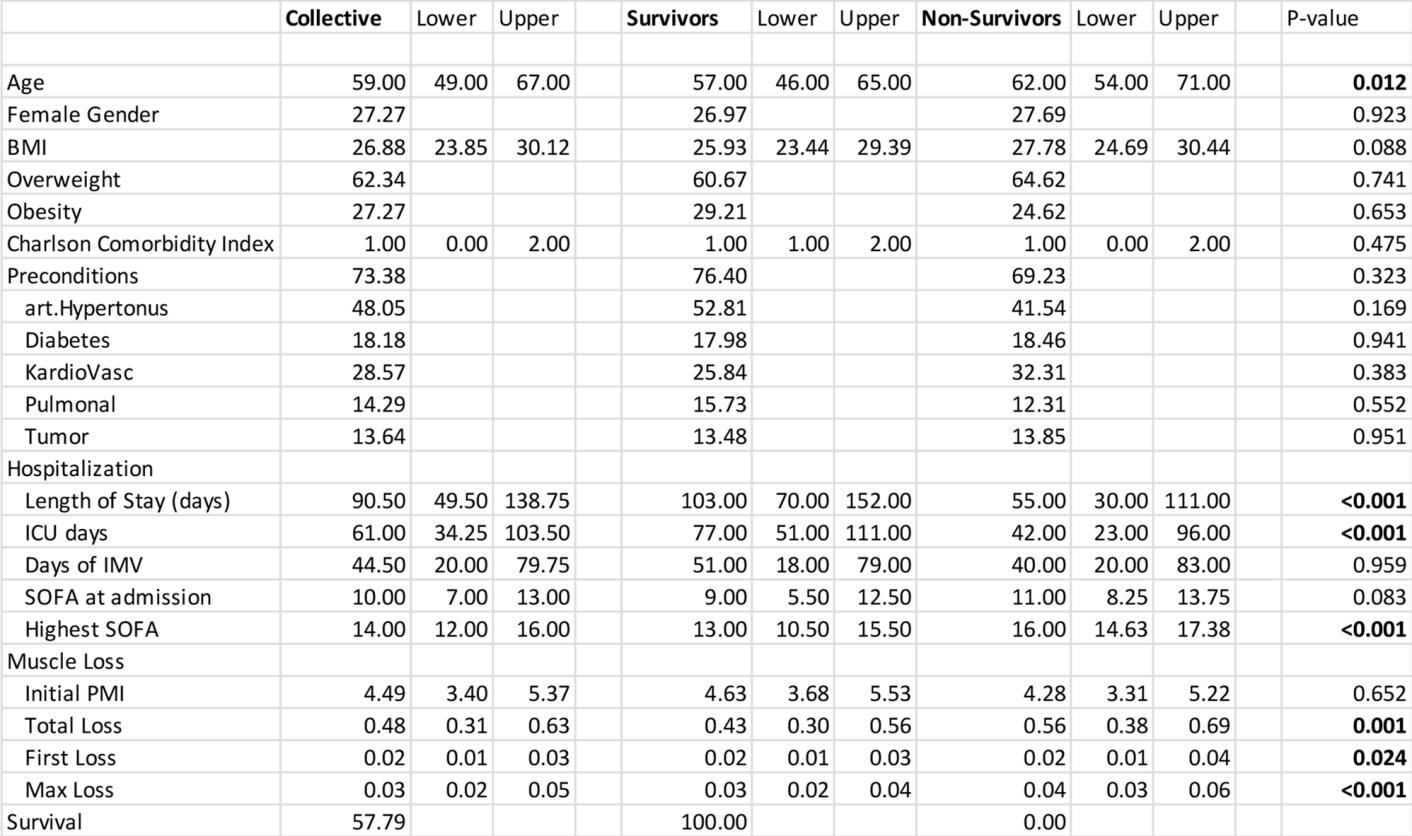
Overview of patient data presented as percentages or medians with interquartile ranges (IQR)

### Frequency of CT imaging

The median number of CT scans per patient was 6 (IQR: 4–8). The overall median interval between scans was 9 days (IQR: 5–16 days). Specifically, the median interval between scans was 8 days (IQR: 5–14 days) from the first to the second scan, 8 days (IQR: 8–13 days) from the second to the third scan, and 10 days (IQR: 6-19.25 days) from the third to the fourth scan.

The majority of CT scans (94.53%) were performed during the ICU stay, while only 5.46% were performed after transfer to a general medical ward.

### Muscle loss during hospitalization

**Fig. 1:**
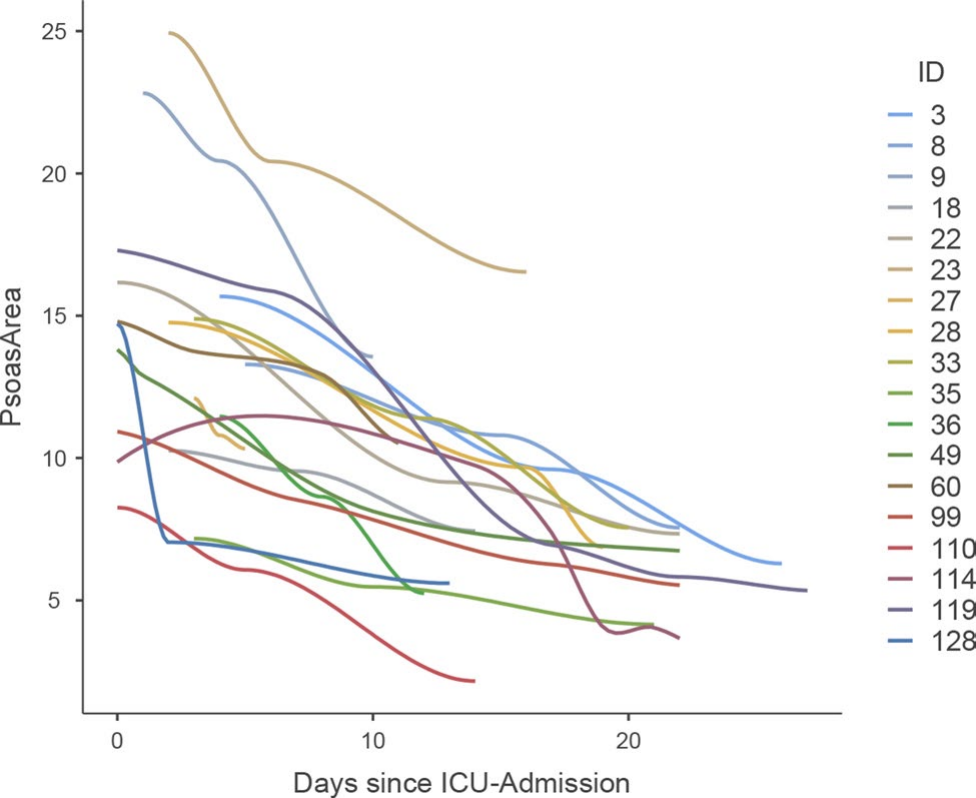
Scatterplot illustrating the muscle wasting in a selection of representative intensive care patients. Each point represents the time of a clinically indicated CT scan and the corresponding psoas muscle area. The graphs clearly illustrate the wide variability in muscle wasting patterns

In both groups, the psoas muscle served as the reference muscle area. The observed cumulative muscle loss exhibited a pattern, characterized by an overall negative trend with varying rates of decline at different time intervals Fig. 1.

**Fig. 2:**
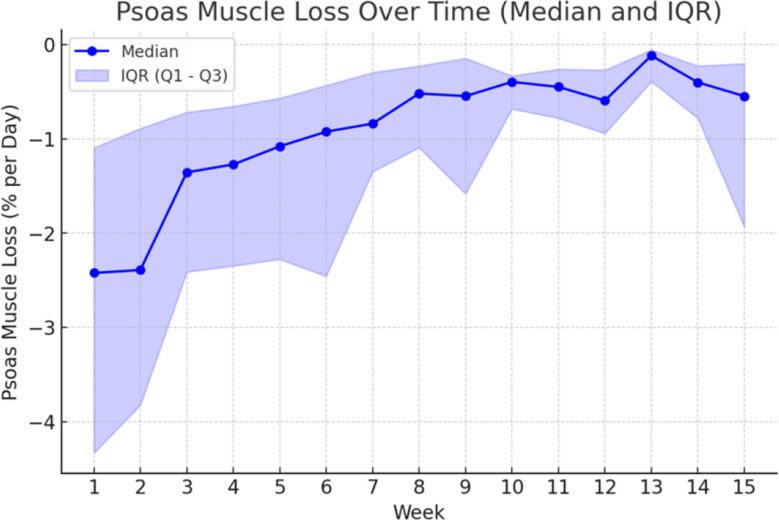
Illustration of muscle loss rates grouped according the week during which they were obtained. The graph clearly illustrates the decreasing loss rates over time, while variability among patients remains high

Muscle loss rates of the whole collective, measured as psoas loss in % per day, were highest in the first two weeks, peaking at -2.42% per day (IQR − 4.33 to -1.10%) in week 1 and − 2.39% (IQR − 3.82 to -0.89%) in week 2, followed by a steady decline. From week 3, the loss rates decreased progressively, reaching − 1.36% per day in week 3 and falling below − 1% per day by week 8 (Fig. 2; Table 2**)**.

**Table 2:**
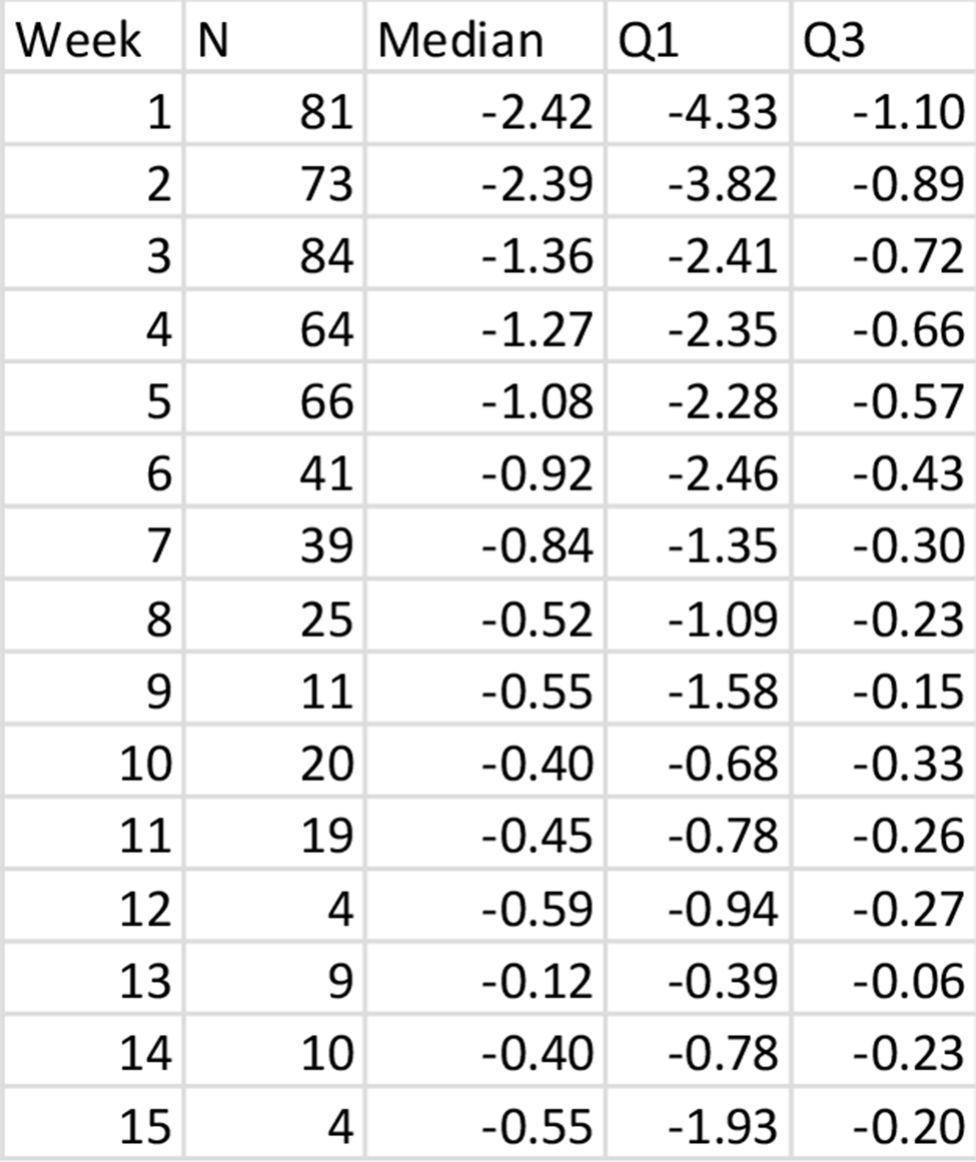
Median muscle decay rates with IQR grouped by the week during which they were obtained. N = number of measurements obtained in the respective week with median muscle decay rate in % per day and IQR (Q1, Q3)

**Fig. 3:**
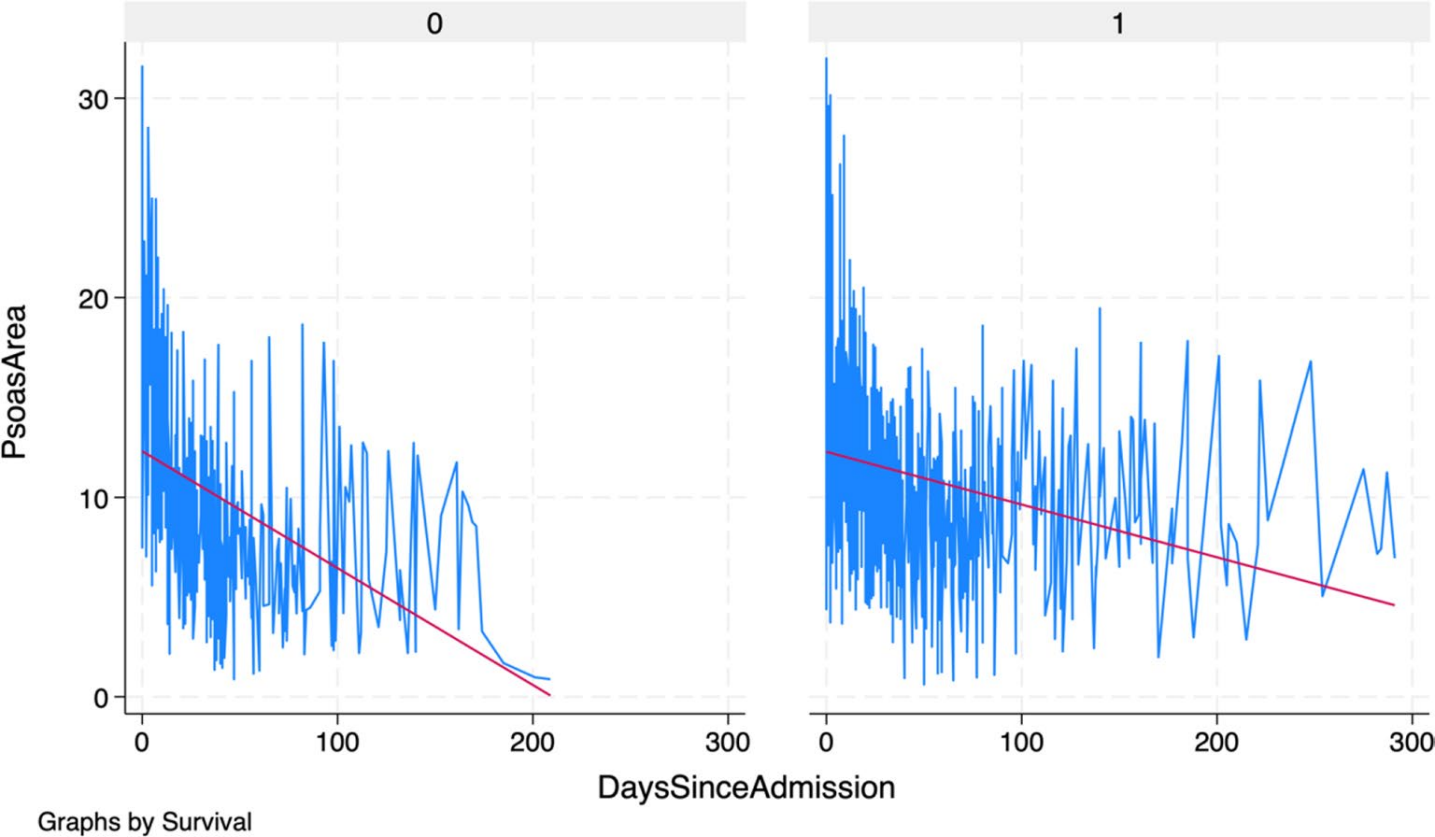
Fitted values of muscle wasting in ICU patients grouped by survival status (0 = non-survivors, 1 = survivors). Non-survivors had a significantly higher rate of muscle loss, as indicated by the red fitted trend line, highlighting the accelerated rate of muscle loss in this group

Patients experienced a median total psoas muscle area (PMA) loss of 48.3 (32.55–64.3)%. The first observed loss rate, occurring between the first consecutive scans, was 1.84 (0.52–3.09)% per day. Maximum loss, between two scans, was 3.47 (2.03–4.92)%. Among the 154 patients, first, maximum and total PMA loss diverged significantly between survivors and non-survivors with 1.60% vs. 2.46% per day (*p* = 0.024), 2.92% vs. 4.36% per day (*p* = 0.03) and 43.10% vs. 56.20% (*p* < 0.001) respectively (Fig. 3; Table 1).

### Muscle loss patterns and survival outcomes

Mixed model analysis revealed a significant reduction of PMA per timepoint of 1.14 cm² (*p* < 0.001), highlighting progressive muscle wasting in ICU patients. Survival was positively associated with greater psoas area (B = 1.4948, *p* = 0.027), suggesting that patients with less muscle decline had better survival outcomes. Moreover, significant differences of muscle area were observed between genders, with significantly less PMA among females across all obtained scans (B = -2.9414, *p* < 0.001). In contrast, age (B = -0.0198, *p* = 0.603) and disease entity (B = -1.0430, *p* = 0.104) were not significantly associated with muscle loss. Similarly, overweight status (B = 0.5722, *p* = 0.469) and sarcopenia (B = 0.2636, *p* = 0.754) had no significant effect on psoas area (Table 3).

**Table 3:**
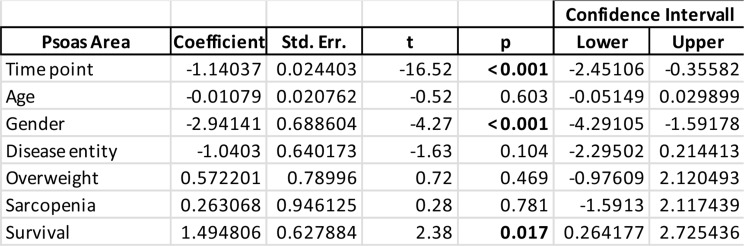
Results from a mixed model analysis assessing the association of various factors with Psoas area. Coefficients represent effect sizes with 95% confidence intervals. Statistically significant values (*p* < 0.05) are bolded

The stepwise backward elimination revealed SMA, VAT, BMI, the highest SOFA score obtained during the stay and the muscle decay as relevant variables. Cox regression analysis showed that VAT (B = 0.009, *p* = 0.001), higher SOFA scores (B = 0.221, *p* = 0.014) and muscle decay (B = 83.609, *p* < 0.001) are significant risk factors for survival, while elevated SMA (B = -0.020, *p* = 0.025) had a protective effect. BMI was not a significant predictor (B = -0.053, *p* = 0.178). Results are compiled in Table 4.

**Table 4:**
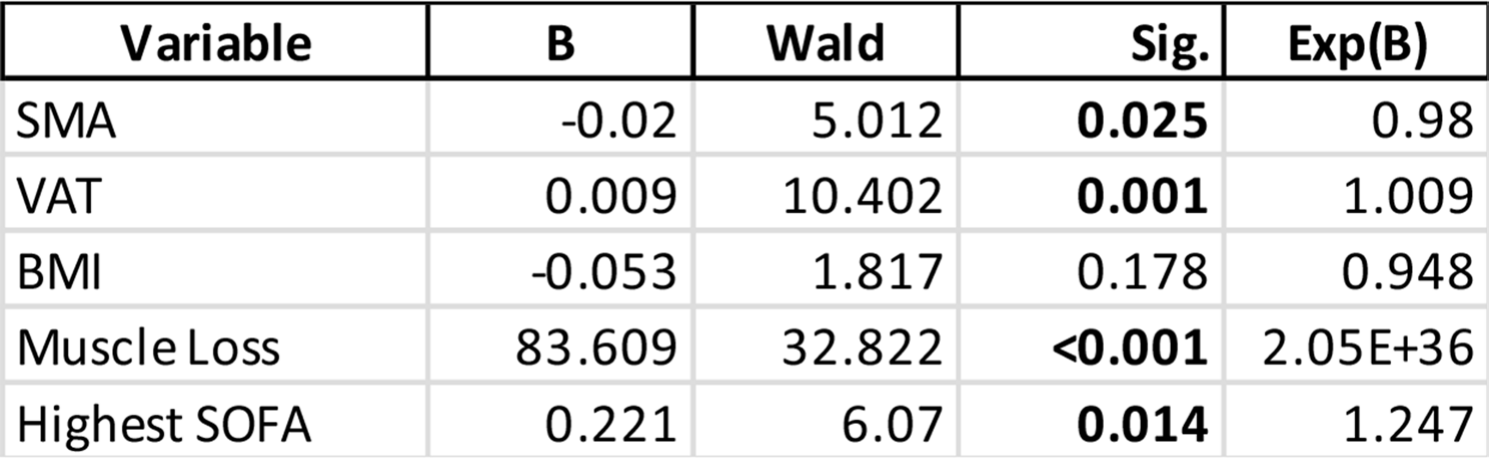
Results of Cox regression for survival in patients experiencing prolonged ICU stays. While high viceral adipose tissue area (VAT), muscle loss and SOFA scores were identified as risk factors for survival. Elevated skeletal muscle area (SMA) was identified as protective for survival. BMI (Body mass Index) was not significant. Significant values (*p* < 0.05) are bolded

**Fig. 4:**
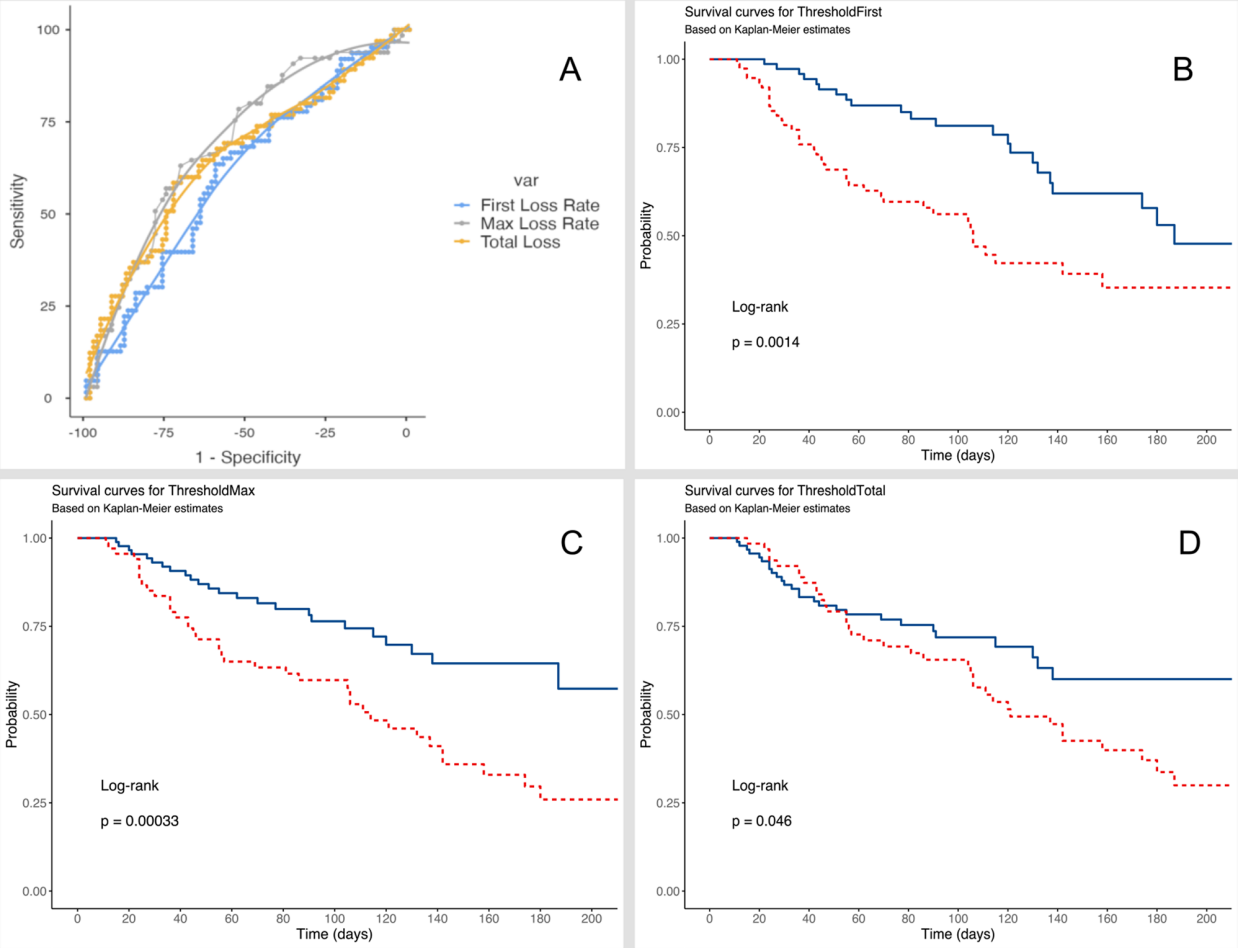
ROC Analysis of Muscle Loss Rates and Total Muscle decay (**A**). Kaplan-Meier Curves of Survival Probabilities based on Muscle Loss Thresholds for First Loss (**B**) with a threshold of 1.97% PMA loss per day, Maximal Loss (**C**) with a threshold of 4.10% PMA decay per day and Total Muscle Decay (**D**) with a threshold of 54.18%

**Fig. 5:**
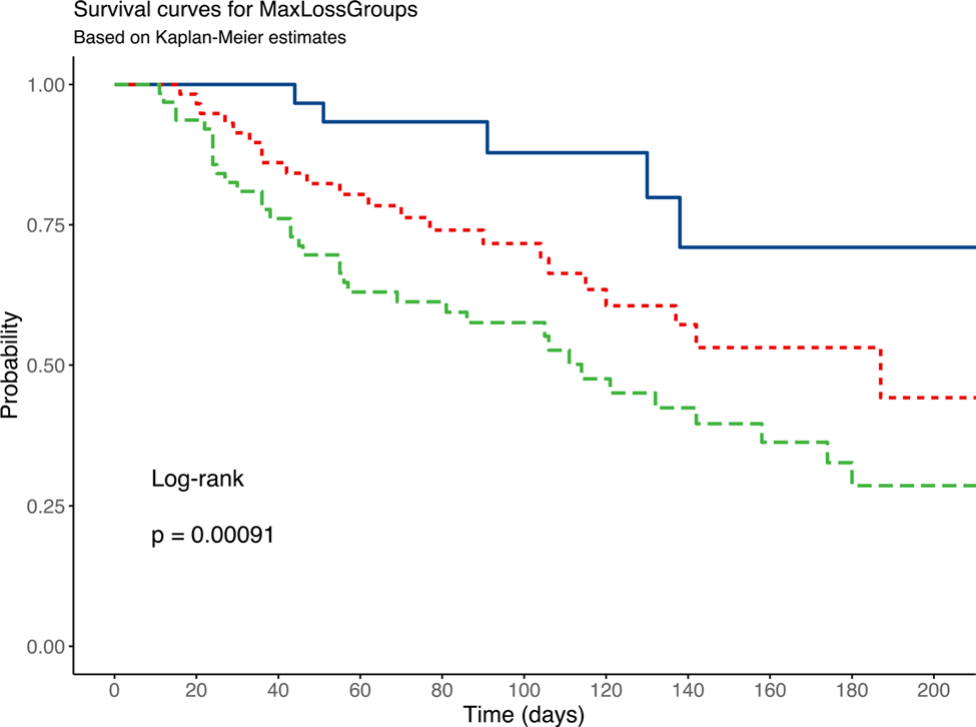
Kaplan Meier-Curves of Maximal Loss Categories for clinical Applicability. Blue = Max. Loss < 2%; Red = Max. Loss 2–4% and Green = Max. Loss > 4%

ROC analysis yielded areas under the curve (AUC) for survival prediction of 0.611, 0.696 and 0.658 for first loss rate, maximum loss rate and total loss rate, respectively. The Youden index identified optimal survival prediction thresholds as follows 1.97% PMA loss per day for first loss rate, 4.10% PMA loss per day for maximum loss rate, and 54.18% total loss. Kaplan-Meier analysis showed statistically significant log-rank values for each of these thresholds, with *p* = 0.046 for total loss, *p* < 0.001 for maximum loss, and *p* = 0.0014 for first PMA loss. Further categorization of maximum loss parameters into ranges of 0–2%, 2–4% and > 4% PMA loss per day revealed a strong correlation between muscle loss and survival outcomes (*p* < 0.001). Patients with a daily muscle loss rate of 0–2% had the highest survival rate at 84.8%, while those in the 2–4% loss group had a significantly lower survival rate at 62.1%. For people with a muscle loss of more than 4% per day, the survival rate dropped significantly to 39.7%. These results are summarized in Figs. 4 and 5.

## Discussion

The present study highlights the dimension of cumulative muscle wasting in a large ICU cohort. Patients exhibited substantial muscle losses with peak decay rates of more than 4% during the first two weeks, muscle deterioration declined continuously thereafter. We observed significant.

differences in muscle decay rates between survivors and non-survivors, e.g. 43.1% vs. 56.2% total loss (*p* < 0.001). Cox regression for survival revealed muscle decay, SMA, VAT and the highest SOFA during hospitalization as significant predictors. ROC analysis identified survival thresholds for initial loss, for peak loss and for total loss,. Stratification of maximum loss rates (0–2%, 2–4%, > 4% per day) showed that higher loss was strongly associated with worse outcomes (*p* < 0.001).

Previous research has consistently documented significant muscle wasting in critically ill patients, with rapid losses occurring during the early days of immobilization. A recent meta-analysis summarized the findings of 3251 critically ill patients, reporting an average daily loss of 2% per day during the first week [7]. While, to our knowledge, we are the first to report muscle decay rates in patients experiencing prolonged hospitalization, the present study confirms and exceeds these observations. Muscle wasting among ICU patients during the first period of admission has been extensively investigated, only few studies have reported long-term muscle loss, all comprising study populations smaller than 100 patients [8, 12, 13, 14, 15]. Borges et al. reported 13.5% and 14.5% muscle loss in two sepsis cohorts (37 and 45 patients) during a 9-10-day ICU stay, with all patients surviving [12, 13]. Hadda et al. found a maximum muscle loss of 15.5% at day 7 in 70 septic patients, with 59 survivors, but did not assess subsequent muscle status [15]. Trung et al. observed 23.49% muscle loss in 70 tetanus ICU patients, including 4 deaths, during stays up to 34 days [14]. Our findings stand out not only due to the long observation periods in patients experiencing prolonged hospitalization patients and the excessive muscle losses, but also for showing that the extent of muscle loss declines over time.

In critical care, traditional tools for assessing muscle size and function, such as anthropometry or handgrip strength, are impractical due to patient non-cooperation and fluid shifts. While more standardized, non-volitional measurement methods have been investigated in several studies, objective assessment of muscle quantity and quality, using tools like bioelectrical impedance analysis (BIA), CT, and ultrasound (US) are rarely integrated into routine clinical practice [16]. In fact, CT offers greater precision than alternatives such as ultrasound, as highlighted by other authors and recommended by guidelines for nutritional status assessment [17–19]. This method is particularly advantageous because body composition can be analyzed within seconds using advanced algorithms, allowing rapid and efficient monitoring without additional patient or financial burden [20]. The robust association between higher maximum loss rates and poorer outcomes, as highlighted by decay stratification into categories of 0–2%, 2–4% and > 4% per day and confirmation of survival differences in Kaplan-Meier analysis, underscores the clinical relevance and feasibility of our monitoring method.

### Limitations

This study provides valuable insights into the dynamics of muscle wasting in ICU patients with COVID-19 ARDS and acute pancreatitis; however, several limitations should be noted. First of all, this is an observational study and therefore the results are limited to being hypothesis-generating only. Second, our study did not control for several confounding variables, including differences in nutritional interventions, use of physiotherapy, onset of sepsis or the use of medications such as corticosteroids and exposure to neuromuscular blocking agents which are known to influence muscle loss and recovery in critically ill patients [21, 22]. Third, considering the retrospective nature, it’s inevitable that there’s a selection bias. This bias likely means that less severe patients are not as represented due to our inclusion criteria. Another major potential source of selection bias is the inclusion of only those patients who underwent repeat CT imaging. Moreover, the heterogeneity in the timing of CT scans may affect the accuracy of the results. In addition, the ten-year study period may reflect shifts in ICU practices over time, and early COVID-19 patients may not have received optimal treatments available today, limiting the study’s contemporaneity. Also, we did not assess the functional outcomes associated with the observed loss rates. By focusing exclusively on patients with COVID-19 ARDS and acute pancreatitis, the results may not represent the dynamics of muscle wasting in other ICU populations. A further limitation is the exclusive focus on the psoas muscle area as a surrogate for total muscle decay. Although the psoas muscle is a commonly used indicator and has been shown to be less susceptible to fluid derangement, it may not fully capture the complexity of muscle loss in different muscle groups.

## Conclusion

In conclusion, this study confirms well-established patterns of rapid and severe muscle loss in ICU patients, while providing new insights into the timing, magnitude and clinical value of muscle wasting dynamics. The identification of survival-related thresholds for muscle wasting rates provides a valuable tool for clinical risk stratification and may help to time targeted interventions. Future research should validate these thresholds in larger cohorts, and investigate tailored strategies to reduce muscle wasting in critically ill patients.

## Electronic supplementary material

Below is the link to the electronic supplementary material.

Supplementary Material 1

Supplementary Material 2

Supplementary Material 3

Supplementary Material 4

## Data Availability

The datasets generated and analyzed during the current study are available from the corresponding author on reasonable request.

## Abbreviations

AI: Artificial Intelligence
ANOVA: Analysis of Variance
AP: Acute Pancreatitis
ARDS: Acute Respiratory Distress Syndrome
BMI: Body Mass Index
CT: Computed Tomography
ICU: Intensive Care Unit
ICUAW: ICU-acquired weakness
PACS: Picture Archiving and Communication System
PMA: Psoas Muscle Area
SAT: Subcutaneous Adipose Tissue
SMA: Skeletal Muscle Area
SMI: Skeletal Muscle Index
SOFA: Sequential Organ Failure Assessment
VAT: Visceral Adipose Tissue
